# Clustering of Metabolic Risk Components and Associated Lifestyle Factors: A Nationwide Adolescent Study in Taiwan

**DOI:** 10.3390/nu11030584

**Published:** 2019-03-09

**Authors:** Wei-Ting Lin, Chun-Ying Lee, Sharon Tsai, Hsiao-Ling Huang, Pei-Wen Wu, Yu-Ting Chin, David W. Seal, Ted Chen, Yu-Ying Chao, Chien-Hung Lee

**Affiliations:** 1 Department of Public Health, College of Health Sciences, Kaohsiung Medical University, Kaohsiung 807, Taiwan; wtlin0123@gmail.com (W.-T.L.); catstar1211@gmail.com (P.-W.W.); kiki13336586@gmail.com (Y.-T.C.); yuyich@kmu.edu.tw (Y.-Y.C.); 2 Department of Global Community Health and Behavioral Sciences, School of Public Health and Tropical Medicine, Tulane University, New Orleans, LA 70112, USA; dseal@tulane.edu (D.W.S.); tchen@tulane.edu (T.C.); 3 Department of Family Medicine, Kaohsiung Medical University Hospital, Kaohsiung Medical University, Kaohsiung 807, Taiwan; cying@ms19.hinet.net; 4 Department of Laboratory Medicine, Kaohsiung Municipal Hsiao-Kang Hospital, Kaohsiung 812, Taiwan; 870718@kmuh.org.tw; 5 Department of Oral Hygiene, College of Dental Medicine, Kaohsiung Medical University, Kaohsiung 807, Taiwan; hhuang@kmu.edu.tw; 6 Research Center for Environmental Medicine, Kaohsiung Medical University, Kaohsiung 807, Taiwan; 7 Department of Medical Research, Kaohsiung Medical University Hospital, Kaohsiung Medical University, Kaohsiung 807, Taiwan

**Keywords:** adolescent, cardiometabolic risk factor, metabolic syndrome, obesity, risk factor clustering, sugar-sweetened beverage, lifestyle factor, Taiwan

## Abstract

Clustering of metabolic syndrome (MetS) risk components in childhood has been linked to a higher risk of diabetes and cardiovascular diseases in adulthood. By using data from the 2010–2011 Nutrition and Health Survey in Taiwan, this study investigated epidemic patterns and correlates for the clustering of MetS risk components. A total of 1920 adolescents aged 12–18 years were included in this study. The MetS diagnostic criteria defined by the Taiwan Pediatric Association (TPA) and International Diabetes Federation (IDF) for adolescents and the criteria defined by the Joint Interim Statement for adults (JIS-Adult) were used to evaluate MetS and its abnormal components. The prevalence of TPA-, IDF-, and JIS-Adult-defined MetS was 4.1%, 3.0%, and 4.0%, with 22.1%, 19.3%, and 17.7%–18.1% of adolescents having high fasting glucose, low high-density lipoprotein cholesterol, and central obesity, respectively. A 0.4-to-0.5-fold decreased risk of having ≥2 MetS abnormal components was detected among adolescents who consumed ≥1 serving/week of dairy products and fresh fruits. Boys who consumed ≥7 drinks/week of soda and girls who consumed ≥7 drinks/week of tea had a 4.6- and 5.2-fold risk of MetS, respectively. In conclusion, our findings revealed significant dimensions of adolescent MetS, including detecting population-specific prevalent patterns for MetS risk components and their clustering, and emphasized on health promotion activities that reduce sugar-sweetened beverage intake.

## 1. Introduction

Metabolic dysfunction, comprising excess abdominal adiposity, elevated serum triglyceride, abnormal lipoprotein cholesterol, increased blood pressure (BP), and high plasma sugar, occurring individually or together, such as metabolic syndrome (MetS), have been linked to an increased risk of type 2 diabetes mellitus (T2DM) and cardiovascular diseases [1]. Epidemiological studies conducted in the United States, Australia, Europe, South America, and Asia have reported that these metabolic dysfunctions have occurred in adolescents [2,3,4,5,6,7,8,9]. Because marked lifestyle improvement for metabolic risk factors can delay or even prevent the development of T2DM and cardiometabolic diseases [10,11,12], active monitoring, identification, and management for adolescents with clustering of MetS risk components become an urgent concern in children.

Anthropometric and metabolic features vary rapidly with age and pubertal growth; thus, universally accepted cutoff points or criteria for MetS diagnosis in adolescents do not exist [13]. The International Diabetes Federation (IDF) definition and the modified guidelines of the National Cholesterol Education Program Adult Treatment Panel III (NCEP ATP III) [14,15] were used to measure the prevalence of adolescent MetS in populations [4,7,8]. Although simultaneously using several different MetS diagnostic criteria would produce somewhat diverse epidemic patterns, it may provide a complete understanding of metabolic dysfunctions and their clustering in youths.

Lifestyle factors, such as obesity, poor nutritional intake, sugar-sweetened beverage (SSB) intake, long screen time, and low physical activity, have been associated with an increased risk of adolescent MetS [16,17,18,19,20]. Because of varying dietary habits, lifestyles, and physical activities in different adolescent populations, investigation of children-specific risk factors or correlates is warranted so as to provide data to develop efficient prevention and intervention activities for adolescent MetS.

Continuous nationwide surveys conducted for monitoring adolescent obesity in Taiwan have indicated that the prevalence of overweight/obesity in male and female adolescents aged 13–18 years has increased from 16.4% and 8.9% in 1991 to 25.2% and 15.2% in 2003 (53.7% and 70.8% increase), respectively [21]. In addition to the increase in obesity and its association with metabolic dysfunctions [14], the clustering of MetS risk factors in childhood becomes a vital health concern for adolescents in Taiwan. In the present study, data from the Nutrition and Health Survey in Taiwan (NAHSIT) was used to investigate and compare the epidemic patterns for the clustering of adolescent MetS risk components in Taiwan using three commonly employed diagnostic criteria and explore the correlates for the clustering of MetS risk components.

## 2. Materials and Methods

### 2.1. Study Design and Population

The NAHSIT is a nationwide, multigroup research program designed to evaluate and monitor the nutritional conditions and health of adults and children in Taiwan. The surveys are comprehensive in that they include integrated nutrition- and health-associated interviews and physical examinations. This study examined the data from the 2010–2011 NAHSIT survey conducted between April 2010 and December 2011 and offered a nationally representative adolescent sample [22].

The target population included all students aged 11–22 years, enrolled in public or private secondary schools in the 2010 academic year in Taiwan, but does not include students enrolled in overseas Chinese schools and special schools. Sampling was performed in 3 stages by using a multistage, stratified clustering sampling technique [22]. First, 358 city districts and townships were categorized into 5 strata according to geographic location and population density (north 1 and 2, center, south, and east strata). Second, the number of students in every school in each stratum was determined, and 6 junior high schools and 4 senior high or vocational schools were randomly chosen from each stratum (30 and 20 schools, respectively) using the method of probabilities proportional to size. Third, 54 and 60 junior and senior students, respectively, were randomly selected from the chosen schools, given the strategy of half male and female and even sample number in each grade. A total of 1965 adolescents and their guardians signed an informed consent declaration for questionnaire interviews and health examinations. The response rates were 87% and 92% in junior and senior high students, respectively [23]. Study subjects were focused on adolescents aged 12–18 years, because there were only one 11-year-old and twenty-one 19- to 22-year-old participants in this survey. We excluded 23 adolescents who had incomplete clinical or dietary data and retained 1920 adolescents for the final analyses.

### 2.2. Questionnaire Data

Survey questionnaires were developed and verified to collect information regarding demographic factors, dietary habits and lifestyle pattern, personal disease history and health conditions, pubertal status, physical activity, and school behavior and emotion [22]. All data were collected by well-trained research staff members. Based on the purpose of this investigation, we only used data obtained from the questionnaires on dietary habits, lifestyle pattern, and physical activity. Personal dietary consumptions were measured using the verified food frequency questionnaire on the categories and frequency of eggs, milk, fresh fruits, meats, fried foods, and SSB in the month before the interview. Daily total calorie intake was estimated by individual data on 24-h dietary recalls and the information offered by the Taiwanese Food and Nutrients Databank [24]. The consumed SSB were soda beverages, sports drinks, coffee drinks, and all types of tea. The frequency of SSB consumption was measured individually for each type of beverage. The daily time spent on sedentary behaviors, including watching television; using computers and electronic games; and reading newspapers, comics, and magazines, was calculated according to regular activities in the previous month. The average time spent on physical exercise daily was calculated according to exercises that lead to an increase in sweat, breathing, and heartbeat in participants in a regular week. The use of tobacco and alcohol were defined as the consumption of tobacco daily or occasionally and the intake of alcohol >1 drink/week, respectively. Data on the stage of pubertal development were obtained using a validated Chinese version of a self-administered rating scale [25].

### 2.3. Anthropometric Parameters

Anthropometric variables, including height, weight, waist circumference (WC), hip circumference (HC), and BP, were measured by a physical examination team at the health center of each selected school. Height and weight were measured to the nearest 0.1 cm and 0.1 kg, respectively. HC and WC were measured to the nearest 0.5 cm by using a flexible and inextensible tap. Average BP was computed by averaging 3 measurements of systolic blood pressure (SBP) and diastolic blood pressure (DBP). Body mass index (BMI) was calculated by weight (kg) divided by the square of height (m).

### 2.4. Clinical Variables

A >8-h fasting blood specimen was collected from each participant by a health examination team in the morning. All blood samples were centrifuged immediately after collection and placed in liquid nitrogen; the samples were then transported to the central laboratory and stored at −70 °C in a refrigerator, waiting for the uniform detection of clinical variables. Using enzymatic procedures with an autoanalyzer, fasting triglyceride (FTG) and high-density lipoprotein cholesterol (HDL-C) levels in the blood specimens were examined. Plasmas were used to identify fasting glucose (FG) levels by using the enzymatic reference method with hexokinase [23].

### 2.5. MetS and Its Risk Components

Three diagnostic criteria, respectively, defined by the Taiwan Pediatric Association (TPA) for adolescents aged 8–18 years, the IDF for adolescents aged 10–18 years, and the Joint Interim Statement for adults (JIS-Adult) MetS were used to investigate abnormal components and MetS [14,26,27]. The definitions for each risk component of MetS are presented in Supplementary Table S1. In brief, central obesity was defined as BMI >95 percentile of gender- and age-specific groups by TPA, WC ≥90 percentile (or adult cutoff if lower) by IDF, and WC ≥90 cm in boys and ≥80 cm in girls by JIS-Adult. Low HDL-C was defined as HDL-C of <40 mg/dL in boys and of <50 mg/dL in girls by TPA and JIS-Adult; low HDL-C was defined as HDL-C <40 mg/dL for adolescents aged 10–15 years and as HDL-C of <40 mg/dL in adolescent boys and <50 mg/dL in adolescent girls aged 16–18 years by IDF. The definitions for the remaining 3 risk components were the same for these 3 criteria: increased BP, SBP ≥130 or DBP ≥85 mmHg (or antihypertensive drug treatment); elevated FTG of ≥150 mg/dL; and high FG of ≥100 mg/dL (or previously diagnosed T2DM). Alternatively, to include all potential adolescent risk components, we defined participants who had any one of the TPA or IDF abnormal components as having a generalized MetS risk component. The TPA, IDF, and generalized method diagnose an adolescent with central obesity plus 2 other risk components as having MetS, whereas JIS-Adult diagnoses an adolescent who has 3 abnormal findings out of 5 as having MetS.

### 2.6. Statistical Analysis

An appropriate sampling weight was assigned to each participant according to the probability of being selected using information on the registered residence area, sex, and age. Survey data modules were used to adjust for sampling weights in all analyses (Stata v15, StataCorp., College Station, TX, USA). Thus, the final results are representative of the nationwide adolescent population of Taiwan. We classified adolescents aged 12–15 and 16–18 years as junior and senior participants, respectively. Means with standard errors and proportions were used to describe the distributions for continuous and categorical variables, respectively. We calculated Cohen’s Kappa coefficient (*k*) to evaluate the agreement of MetS identification between different diagnostic criteria. The *k* values of 0.41–0.60, 0.61–0.80, and >0.80 can be interpreted as moderate, good, and very good agreement, respectively [28]. The principal component analysis and a 2-dimensional biplot were used to illustrate the correlation and clustering of MetS risk components among gender- and age-specific groups as well applied in other studies [29,30,31]. High-correlated risk components were grouped into a cluster in both genders. We used multivariable logistic regression models to evaluate the gender-specific relationship between lifestyle factors and the clustering of MetS-associated risk components. Potential confounders considered in the multivariable models included residence area, age, puberty status, daily total energy intake, cigarette smoking, alcohol drinking, and, where appropriate, total SSB consumption and physical activity.

## 3. Results

The distributions of demographic factors, dietary patterns, physical activity, and substance use among both genders and junior and senior students are shown in Table 1. Adolescent boys had higher intakes of milk, fresh fruit, SSB (including soda and sports drinks), and total calories and a greater proportion of sedentary behavior, physical exercise, and cigarette smoking than adolescent girls. Differences in the distributions for particular dietary patterns, physical activity, and substance use were also observed between junior and senior students.

Gender differences in the distribution and prevalence of MetS risk components among junior and senior adolescents are presented in Table 2. In both age groups, boys had higher mean SBP and FG values than girls (all *p* ≤ 0.001), whereas girls had greater mean HDL-C values than boys (all *p* ≤ 0.005). According to the BMI-defined TPA criteria, the prevalence of central adiposity was higher in boys than in girls (23.9% vs. 11.9% in juniors and 19.8% vs. 13.6% in seniors), whereas according to the WC-defined IDF/JIS-Adult criteria, the prevalence was higher in girls than in boys (17.9% vs. 12.5% in juniors and 27.2% vs. 18.3% in seniors). Among all adolescents, the TPA/JIS-Adult criteria identified a heterogeneous distribution of low HDL-C between genders (27.1% and 12.4% for girls and boys, respectively, *p* < 0.001). The prevalence of increased BP and high FG in junior and senior boys was higher than that in junior and senior girls.

The prevalence of adolescent MetS defined by TPA and IDF adolescent criteria and JIS-Adult criteria is shown in Table 3. According to the TPA criteria, 7.34% of adolescents had a high BMI and one abnormal component of MetS, with a higher prevalence, was observed in juniors than in seniors (8.06% vs. 6.31%). In juniors, the prevalence of MetS (3.74%–5.48%) defined by the TPA criteria was greater than that defined by the IDF criteria (1.76%–3.59%) but similar to that defined by the JIS-Adult criteria (4.32%–4.38%) in both genders, although the corresponding prevalence was comparable across the three diagnostic criteria in seniors. A very good agreement for MetS diagnosis was identified between the TPA and JIS-Adult criteria in junior and senior boys and girls (*k*, 0.836–0.969). However, only a moderate-to-good agreement was identified between the TPA and IDF criteria in juniors (*k*, 0.519–0.783). The overall MetS prevalence for adolescent boys and girls was determined to be 5.03% and 3.34%, respectively, using either the TPA or IDF criteria.

**Figure 1:**
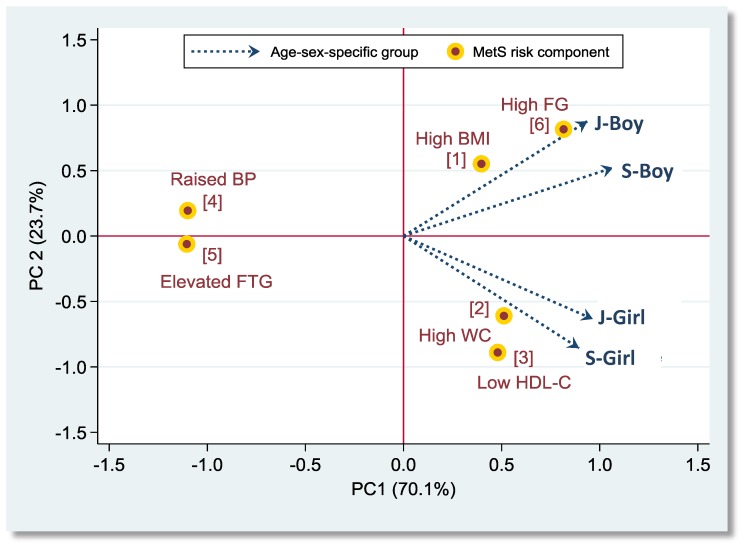
Two-dimensional biplot for risk components of adolescent metabolic syndrome (MetS) and gender-age-specific adolescent groups. **Note**: Red circles denote six adolescent MetS-associated risk components defined by the TPA-IDF generalized criteria. Arrows denote four gender-age-specific adolescent groups (J-Boy, junior boys; J-Girl, junior girls; S-Boy, senior boys; S-Girl, senior girls). The percentages indicate the amount of variance accounted for by principal components PC1 and PC2. Total explained variance from the first two components is 93.8%.

The clustering of six risk components of adolescent MetS in boys and girls is illustrated in Figure 1. High BMI and high FG were associated and clustered in junior and senior boys (aggregately in the first quadrant), whereas high WC and low HDL-C were correlated and grouped in junior and senior girls (aggregately in the fourth quadrant). Boys and girls with a clustering of risk components were grouped and investigated for corresponding risk factors (Table 4).

The relationship between dietary pattern and the clustering of TPA–IDF generalized criteria-defined risk components for MetS is displayed in Table 4. The consumption of ≥1 serving/week of dairy products (adjusted odds ratio (aOR) = 0.5) and fresh fruit (aOR = 0.4) was associated with a lower likelihood of contracting ≥2 abnormal components of MetS in both girls and boys. Similar patterns were observed among adolescent boys with high BMI or high FG.

The association of SSB intake and physical activity with the clustering of MetS risk components in adolescents is presented in Table 5. In boys, an association was identified between coffee intake and high BMI as well as between sports drinks intake and high FG. Excessive soda consumption (≥7 drinks/week) was associated with ≥2 abnormal components of MetS and developing MetS. In girls, the clustering of these two categories of risk components was correlated with tea intake. No significant correlation between factors associated with physical activity and the clustering of MetS risk components was identified.

Table 6 presents the associations of significant dietary habits with the clustering of MetS risk components adjusted for independent variables among adolescents. Because of the strong correlation between the intake of dairy products and the intake of fresh fruit, these two factors were combined into one variable. In boys, the intakes of ≥1 drinks/week of coffee and ≥7 drinks/week of sports drinks were associated with high BMI or high FG (aOR = 1.7 and 2.7, respectively). Compared with boys who consumed <1 drink/week, boys who consumed ≥7 drinks/week of soda had a 3.5- and 4.6-fold higher risk of ≥2 abnormal components of MetS and MetS, respectively. In girls, MetS was correlated with the consumption of 1–6 drinks/week and ≥7 drinks/week of tea (aOR = 2.8 and 5.2, respectively).

## 4. Discussion

Given the insight into the nationwide pattern for the clustering of MetS risk components in adolescents using three common diagnostic criteria, this study identified that high FG, low HDL-C, and central obesity are prevalent in the adolescent population of Taiwan, in that the overall prevalence was found to be 22.1%, 19.3% (TPA/JIS-Adult criteria), and 17.7%–18.1%, respectively. The clustering of metabolic risk factors is not regarded as a disease and is easily overlooked during all life stages. Because epidemiological studies have identified such gathering of metabolic dysfunctions in childhood predicting adult MetS, T2DM, and cardiovascular diseases [32,33], early identification and management for pediatric MetS becomes a vital focus in child healthcare. Central obesity is a common metabolic risk factor. However, our data additionally focus on the issues of high FG and low HDL-C in Taiwanese adolescents, with a higher emphasis on high FG in boys (prevalence, 27.5%) and low HDL-C in girls (prevalence, 27.1%).

In the assessment of adolescent central obesity, the TPA criteria used the cutoff points of BMI >95 percentile for gender- and age-specific groups [26], whereas the IDF criteria used the cutoff points of WC >90 percentile, and JIS-Adult criteria used the cutoff points of WC of ≥90 cm and ≥80 cm for boys and girls, respectively [14,27]. In this investigation, the TPA-defined prevalence of central obesity was higher in boys than in girls (23.9% vs. 11.9% in juniors and 19.8% vs. 13.6% in seniors); however, the IDF- and JIS-Adult-defined prevalence was higher in girls than in boys (17.9% vs. 12.5% in juniors and 27.2% vs. 18.3% in seniors). This raises the argument that which bodyweight-associated indicator is better. From juniors to seniors, the change in the prevalence for central obesity was in the same direction as that for the IDF- and JIS-Adult criteria (5.8% and 9.3% increases in boys and girls, respectively) but in the opposite direction for the TPA criteria (4.1% decrease in boys and 1.7 increase in girls). WC-defined cutoff points provide a stable method for identifying central obesity in adolescents.

The TPA and JIS-Adult used a less stringent criterion for determining low HDL-C among adolescent girls aged 10–15 years than did the IDF criteria (<50 and <40 mg/dL, respectively) [14,26,27], thus showing a higher prevalence of low HDL-C in juniors versus seniors (31.3% vs. 5.8%). Nevertheless, given that the difference is substantial, it implies a sizeable number of girls having HDL-C levels between 40 and 50 mg/dL. Given that an active lifestyle for improving metabolic risk components can postpone or circumvent T2DM and cardiometabolic diseases [10,11], generalized measures that screen adolescents who meet the TPA or IDF criteria for all risk components are warranted to be used.

In this study, the prevalence of high FG was determined to be 22.1%, with the prevalence among boys being higher than that among girls (27.5% vs. 16.1%). Although this prevalence was higher than those identified among Hispanic (15.2%), White (14.8%), Black (9.4%), and Korean (11.4%) populations [6,7], a comparable prevalence (23.1%) was identified in the United States 2011–2016 National Health and Nutrition Examination Survey (boys vs. girls, 31.6% vs. 13.5%) [9]. The variations in the prevalence of high FG across populations may be attributable to variations in the study region, ethnicity, genetic susceptibility, subject age, and study period. In this survey, boys exhibited higher SSB consumption and BMI values than did girls, and SSB intake (especially from sports drinks and coffee) was linked to the clustering of high BMI and high FG. This might partly explain the higher prevalence of high FG in boys than in girls.

The MetS prevalence in adolescents was reported to be 3.7%–8.6% in the United States, 2.1%–5.7% in Korea, and 1.4%–3.4% in China by nationwide epidemiological surveys conducted during 2001 and 2016 using the diagnostic criteria of modified NCEP ATP III and IDF [6,7,8,9]. Comparable to those for Korean and Chinese adolescents [7,8], this study correspondingly identified 4.1% and 3.0% of TPA- and IDF-defined prevalence of MetS in adolescents, with the TPA criteria being more sensitive in identifying this syndrome in both girls and boys (TPA vs. IDF: 5.0% vs. 3.9% in boys and 3.1% vs. 2.0% in girls).

The Kappa statistics exhibited a very good agreement for MetS diagnosis between the TPA and JIS-Adult criteria (*k*, 0.836–0.925 in juniors and 0.966–0.969 in seniors; Table 3). Based on the TPA method, which uses high BMI to define central obesity, adolescents are diagnosed with MetS if they have central obesity plus two other risk components. By contrast, the JIS-Adult method uses high WC to define central obesity, and the criteria for the diagnosis of MetS among adolescents is exhibiting three out of five abnormal findings [26,27]. The high agreement in MetS diagnosis between these two methods prompts two considerations. First, the majority of adolescents who meet the TPA criteria also meet the criteria of adult MetS. Second, adolescents with three or more of the abnormal components specified by the JIS-Adult method for diagnosing MetS have a greater likelihood of having a high BMI. Because of the nature of a cross-sectional study, the 2010–2011 NAHSIT survey only offers a snapshot of MetS risk components for the study population in a specific time period. Agreement between the two criteria for MetS diagnosis must be validated in other adolescent populations.

The prevalence for adolescents having TPA–IDF-defined central adiposity and any one risk component was found to be 2.2-fold higher than that for the TPA–IDF generalized MetS (9.42% vs. 4.23%, Table 3). In clinical reports, the clustering of MetS risk components has been more underscored over the need to ascertain pediatric MetS [34]. Our findings accentuate that the monitoring of adolescent MetS should not ignore the children who have the potential to develop MetS.

Several adult cohort studies have revealed the beneficial effects of dairy product intake on MetS and glycemic disorders [35,36]. Our results support such findings, in that a reverse relationship between the intake of ≥1 serving/week of dairy product and ≥2 risk components of MetS was identified in both girls and boys. Similarly, adolescents who consumed ≥1 serving/week of fresh fruit had less than 50% risk of having ≥2 MetS abnormal components in this study (aOR = 0.4 in both girls and boys). A meta-analysis of 26 observational studies also suggested that the consumption of fruit is inversely associated with MetS [37].

Sucrose is a disaccharide of 50% glucose and 50% fructose that is hydrolyzed to saccharide in the body. Compared with glucose, fructose, either from sucrose or high-fructose corn syrup, is considered to be a significant driver of developing fatty liver disease, hypertriglyceridemia, and MetS by inducing liver de-novo lipogenesis [38,39,40,41]. Large-scale prospective and cross-sectional studies conducted in the United States, Australia, China, and Taiwan have comparably linked the high amount of SSB intake to elevated risks of abdominal obesity, overall cardiometabolic risk, and MetS in adolescents [3,4,42,43], whereas some investigations have offered different findings [43,44]. Our findings support a positive association between SSB consumption and pediatric MetS, in that a 4.6- and 5.2-fold risk of MetS was identified among boys who consumed ≥7 drinks/week of soda and girls who consumed ≥7 drinks/week of tea, respectively. In addition, high frequent consumptions of sports drinks and coffee were linked to the clustering of high BMI and high FG in boys. In human intervention studies, an enhanced level of visceral fat accumulation, triglycerides, and total cholesterol has been detected among people with high SSB consumption [38].

In this study, special schools include subsidiary schools and special education schools. The proportion of students enrolled in overseas Chinese schools and special schools among the general student population is <0.10% and 0.31%, respectively [45]. Because relatively few students were excluded in the sampling population, the potential selection bias should be limited.

The main strengths of this study are that a large-scale nationally representative sample was used to investigate the clustering of adolescent MetS risk components and evaluate the correlates controlling for several potential confounders. This study has several limitations. First, the clustering of risk components was assessed using several diagnostic methods because currently, no standard criteria exist for MetS in adolescents. Second, the cross-sectional nature of this study restricted any causal evaluation between correlates and risk component clustering. Third, because blood samples were collected and examined from only one time-point, this survey offered merely a snapshot of MetS risk components for the study population.

## 5. Conclusions

This study revealed the noticeable prevalence of high FG, low HDL-C, and central obesity and their clustering in Taiwanese adolescents and demonstrated the reverse association of the intakes of dairy product and fresh fruit and the positive association of SSB intake with risk component clustering. Our findings reveal significant dimensions of MetS in adolescents, including detecting population-specific prevalent patterns for MetS risk components and their clustering, and lend support to the emphasis placed on health promotion activities for reducing SSB intake.

## Figures and Tables

**Table 1:** Distributions of demographic parameters and lifestyle factors in adolescents, Taiwan.

Factor/Category	Total	Gender		*p* Value ^2^	Grade ^1^		*p* Value ^2^
		Male	Female		Junior	Senior	
Study participants, number	1920	949	971		990	930	
**Design-adjusted distribution ^3^**							
**Demographic factors**							
Age (year), mean ± SE	15.0 ± 0.1	15.0 ± 0.1	15.0 ± 0.1	0.980	13.7 ± 0.1	16.8 ± 0.03	<0.001
Study area, %							
North	46.0	46.5	45.5	0.864	47.6	43.8	0.339
Center	25.8	25.1	26.5		25.6	25.9	
South	25.7	25.8	25.6		24.4	27.5	
East	2.5	2.7	2.4		2.4	2.7	
**Postpuberty, %**	85.4	74.9	97.0	<0.001	77.4	96.6	<0.001
**Lifestyle factors**							
**Dietary pattern (binary), %**							
Egg, ≥1 serving/day	40.2	40.9	39.4	0.580	37.5	43.9	0.017
Milk, ≥1 serving/day	22.5	25.2	19.5	0.016	26.2	17.3	<0.001
Fresh fruit, ≥1 serving/day	29.0	33.6	24.1	0.001	34.7	21.3	<0.001
Meats, ≥1 serving/day	18.7	18.0	19.5	0.486	17.4	20.6	0.128
Fried food, ≥1 serving/week	42.5	43.0	42.0	0.738	41.9	43.4	0.599
Sugar-sweetened beverage,≥1 drink/week	88.7	90.9	86.1	0.006	87.7	90.0	0.154
Soda, ≥1 drink/week	28.4	36.1	19.8	<0.001	28.8	27.9	0.737
Sport drink, ≥1 drink/week	28.4	37.3	18.4	<0.001	28.4	28.4	0.991
Coffee, ≥1 drink/week	17.6	17.1	18.2	0.596	14.8	21.6	0.002
Tea, ≥1 drink/week	64.6	65.2	63.9	0.629	59.3	72.2	<0.001
Total calories intake (kcal), mean ± SE	2354.8 ± 27.3	2626.5 ± 37.1	2056.3 ± 37.8	<0.001	2304.5 ± 38.4	2425.5 ± 37.7	0.025
**Physical activity (binary), %**							
Sedentary behavior, ≥60 min/day	55.5	59.9	50.5	0.001	51.9	60.5	0.002
Physical exercise, ≥30 min/day	38.2	48.7	26.5	<0.001	34.7	43.1	0.002
**Substance use (binary), %**							
Alcohol drinking	12.5	12.8	12.1	0.721	8.4	18.2	<0.001
Cigarette smoking	5.1	6.8	3.2	0.007	3.5	7.4	0.003

^1^ Junior and senior denote adolescents aged 12–15 and 16–18 years, respectively. ^2^ *p* values for the mean or proportional differences between genders or between grades. ^3^ Displayed data was adjusted for study design and sample weight.

**Table 2:** Distributions and prevalence of MetS risk components defined by TPA and IDF adolescent criteria and JIS-Adult criteria in adolescents, Taiwan.

MetS Components		Junior (12–15 years)				Senior (16–18 years)				All		
	Total	Male	Female	*p* ^1^	Total	Male	Female	*p* ^1^	Total	Male	Female	*p* ^1^
**Distribution, aMean ± SE ^2^**												
WC (cm)	74.8 ± 0.4	75.4 ± 0.6	74.2 ± 0.5	0.162	78.3 ± 0.4	79.7 ± 0.6	76.8 ± 0.4	<0.001	76.3 ± 0.3	77.2 ± 0.4	75.2 ± 0.3	<0.001
HDL-C (mg/dL)	55.1 ± 0.5	53.4 ± 0.7	56.8 ± 0.9	0.005	55.2 ± 0.4	51.2 ± 0.6	59.7 ± 0.7	<0.001	55.1 ± 0.4	52.4 ± 0.5	58.1 ± 0.6	<0.001
SBP (mmHg)	104.4 ± 0.4	108.6 ± 0.6	99.7 ± 0.5	<0.001	104.3 ± 0.4	109.7 ± 0.5	98.4 ± 0.5	<0.001	104.4 ± 0.3	109.2 ± 0.4	99.1 ± 0.4	<0.001
DBP (mmHg)	60.6 ± 0.3	61.1 ± 0.5	60.1 ± 0.5	0.180	60.0 ± 0.3	60.0 ± 0.4	59.9 ± 0.4	0.865	60.3 ± 0.2	60.6 ± 0.3	60.0 ± 0.3	0.228
FTG (md/dL)	71.6 ± 1.4	71.9 ± 2.4	71.2 ± 1.8	0.846	72.5 ± 1.2	74.8 ± 1.7	69.9 ± 1.7	0.043	71.9 ± 1.0	73.1 ± 1.6	70.6 ± 1.2	0.227
FG (mg/dL)	95.2 ± 0.3	96.3 ± 0.4	94.0 ± 0.4	<0.001	94.8 ± 0.5	96.4 ± 0.5	92.9 ± 1.0	0.001	95.0 ± 0.3	96.3 ± 0.3	93.6 ± 0.5	<0.001
**Prevalence, %**												
Central obesity ^3^												
TPA (high BMI)	18.3	23.9	11.9	0.003	16.8	19.8	13.6	0.046	17.7	22.2	12.6	<0.001
IDF (high WC)	15.0	12.5	17.9	0.026	22.5	18.3	27.2	0.002	18.1	14.9	21.8	<0.001
JIS-Adult (high WC)	15.0	12.5	17.9	0.026	22.5	18.3	27.2	0.002	18.1	14.9	21.8	<0.001
Low HDL-C												
TPA/JIS-Adult	21.3	12.5	31.3	<0.001	16.5	12.3	21.2	0.001	19.3	12.4	27.1	<0.001
IDF	9.4	12.5	5.8	0.005	16.5	12.3	21.2	0.001	12.3	12.4	12.2	0.484
Increased BP	3.8	6.4	1.0	<0.001	2.3	4.0	0.4	0.003	3.2	5.4	0.8	<0.001
Elevated FTG	2.8	2.9	2.6	0.454	3.2	4.0	2.4	0.219	3.0	3.3	2.5	0.215
High FG	24.9	29.1	20.1	0.043	18.2	25.2	10.5	<0.001	22.1	27.5	16.1	<0.001

**Abbreviations:** TPA, Taiwan Pediatric Association; IDF, International Diabetes Federation; JIS-Adult, Joint Interim Statement of MetS for adults; MetS, metabolic syndrome; WC, waist circumference; SBP, systolic blood pressure; DBP, diastolic blood pressure; HDL-C, high-density lipoprotein cholesterol; FTG, fasting triglyceride; FG, fasting glucose; BMI, body mass index; BP, blood pressure.^1^ *p* values for the mean or proportional differences between genders were obtained adjusted for area, age, and puberty.^2^ aMean displays the estimated prediction when the covariates were set as mean values.^3^ Central obesity was determined by TPA-defined gender-age-specific BMI criteria, IDF-defined >90th percentile of WC, and JIS-Adult-defined WC ≥90 cm in male and ≥80 cm in female, respectively.

**Table 3:** Prevalence of MetS defined by TPA and IDF adolescent criteria and JIS-Adult criteria in adolescents, Taiwan.

MetS/Category		Junior (12–15 years)				Senior (16–18 years)				All		
	Total	Male	Female	*p* ^1^	Total	Male	Female	*p* ^1^	Total	Male	Female	*p* ^1^
Metabolic disorders, %												
**TPA criteria**												
High BMI + 1aC	8.06	10.33	5.51	0.079	6.31	6.59	6.00	0.998	7.34	8.80	5.71	0.148
MetS (high BMI + ≥2aC)	4.66	5.48	3.74	0.521	3.35	4.37	2.21	0.089	4.12	5.03	3.11	0.169
**IDF criteria**												
High WC + 1aC	5.13	5.41	4.81	0.765	8.35	6.17	10.75	0.009	6.45	5.72	7.27	0.057
MetS (high WC + ≥2aC)	2.73	3.59	1.76	0.161	3.41	4.37	2.35	0.110	3.01	3.91	2.01	0.040
MetS Kappa (TPA vs. IDF)	0.690 *	0.783 *	0.519 *		0.990 *	1.000 *	0.969 *		0.808 *	0.871 *	0.686 *	
**JIS-Adult criteria**												
2aC	10.02	9.31	10.82	0.620	10.07	9.00	11.25	0.210	10.04	9.18	11.00	0.251
MetS (≥3aC)	4.35	4.38	4.32	0.967	3.57	4.67	2.35	0.074	4.03	4.50	3.50	0.403
MetS Kappa (TPA vs. JIS-adult)	0.874 *	0.836 *	0.925 *		0.967 *	0.966 *	0.969 *		0.907 *	0.887 *	0.938 *	
**TPA-IDF-defined central adiposity + 1aC, %**	9.82	10.33	9.24	0.786	8.84	6.86	11.02	0.019	9.42	8.91	9.98	0.137
**TPA-IDF generalized MetS ^2^, %**	**4.80**	**5.48**	**4.03**	0.602	**3.41**	**4.37**	**2.35**	0.110	**4.23**	**5.03**	**3.34**	0.224

**Abbreviations:** TPA, Taiwan Pediatric Association; IDF, International Diabetes Federation; JIS-Adult, Joint Interim Statement of MetS for adults; MetS, metabolic syndrome; BMI, body mass index; WC, waist circumference; 1aC, 1 abnormal component; 2aC, 2 abnormal components; 3aC, 3 abnormal components; *, *p* < 0.05.^1^ *p* values for the proportional differences between genders were obtained adjusted for area, age, and puberty.^2^ Generalized to include adolescents who meet the TPA or IDF MetS criteria.

**Table 4:** Adjusted odds ratio (aOR) of the clustering of MetS risk components associated with the dietary pattern in adolescents, Taiwan.

Factor/Category	Male					Female				
	High BMI	High FG	High BMI or High FG	≥2 MetS-aC	MetS	High WC	Low HDL-C	High WC or Low HDL-C	≥2 MetS-aC	MetS
	aOR (95%CI)	aOR (95%CI)	aOR (95%CI)	aOR (95%CI)	aOR (95%CI)	aOR (95%CI)	aOR (95%CI)	aOR (95%CI)	aOR (95%CI)	aOR (95%CI)
**Total calories intake ^1^**										
≥2500 vs. <2500 kcal/day	0.8 (0.5–1.4)	1.4 (0.8–2.5)	1.3 (0.8–2.1)	0.7 (0.4–1.4)	1.1 (0.4–2.8)	1.5 (0.7–2.8)	1.3 (0.6–2.6)	1.5 (0.8–2.8)	1.4 (0.6–3.2)	0.6 (0.1–3.3)
**Dietary pattern ^2^**										
**Egg**										
1–6 vs. <1 serving/week	1.2 (0.5–2.7)	1.0 (0.5–2.3)	1.4 (0.7–2.9)	1.3 (0.5–3.6)	1.1 (0.2–5.2)	1.3 (0.5–3.6)	1.3 (0.5–3.4)	1.6 (0.6–4.3)	0.8 (0.3–2.3)	1.9 (0.2–15.9)
≥7 vs. <1 serving/week	1.2 (0.5–2.7)	0.9 (0.4–2.0)	1.2 (0.6–2.5)	1.4 (0.5–4.2)	1.3 (0.3–6.5)	2.2 (0.8–6.1)	1.4 (0.5–3.7)	2.1 (0.8–5.6)	1.5 (0.5–4.6)	2.4 (0.3–21.3)
**Dairy products**										
≥1 vs. <1 serving/week	0.6 (0.4–0.9)	0.7 (0.5–1.1)	0.6 (0.4–0.9)	0.5 (0.3–0.8)	0.6 (0.3–1.5)	0.7 (0.4–1.2)	1.0 (0.6–1.7)	0.9 (0.6–1.5)	0.5 (0.2–0.9)	0.6 (0.2–2.0)
**Fresh fruit**										
≥1 vs. <1 serving/week	0.5 (0.3–0.8)	0.7 (0.5–1.1)	0.6 (0.4–0.9)	0.4 (0.2–0.7)	0.6 (0.2–1.5)	0.7 (0.4–1.2)	1.1 (0.6–1.8)	1.0 (0.6–1.6)	0.4 (0.2–0.9)	0.5 (0.2–1.8)
**Meat**										
1–6 vs. <1 serving/week	1.0 (0.6–1.7)	0.8 (0.5–1.4)	0.9 (0.6–1.4)	1.5 (0.8–2.9)	2.1 (0.8–5.8)	1.2 (0.8–1.9)	1.0 (0.6–1.5)	1.0 (0.6–1.5)	1.3 (0.7–2.3)	1.5 (0.5–4.4)
≥7 vs. <1 serving/week	1.4 (0.7–2.5)	1.1 (0.6–1.9)	1.2 (0.7–2.1)	2.0 (0.9–4.2)	1.9 (0.6–6.0)	1.1 (0.6–2.0)	0.9 (0.5–1.7)	0.9 (0.5–1.5)	1.6 (0.8–3.1)	0.5 (0.1–2.8)
**Fried food**										
1–6 vs. <1 serving/week	0.7 (0.5–1.1)	0.8 (0.6–1.2)	0.8 (0.6–1.2)	0.9 (0.5–1.4)	0.7 (0.3–1.6)	1.0 (0.7–1.5)	1.1 (0.7–1.6)	1.0 (0.7–1.4)	1.2 (0.8–2.0)	1.3 (0.5–4.0)
≥7 vs. <1 serving/week	0.8 (0.3–2.1)	1.1 (0.5–2.5)	0.8 (0.3–1.8)	0.8 (0.3–2.5)	1.3 (0.3–4.7)	0.5 (0.2–1.8)	0.5 (0.2–1.7)	0.5 (0.2–1.4)	0.2 (0.02–1.3)	NA

**Abbreviations**: MetS, metabolic syndrome; FG, fasting glucose; BMI, body mass index; WC, waist circumference; HDL-C, High-density lipoprotein cholesterol; ≥2 MetS-aC, central adiposity + ≥1 abnormal components (aC) of MetS (MetS and its abnormal components were defined by TPA-IDF generalized criteria); NA, non-applicable due to limited sample size.^1^ aOR were adjusted for area, age, puberty, physical activity, cigarette smoking, and alcohol drinking. ^2^ aOR were adjusted for area, age, total energy intake, puberty, physical activity, cigarette smoking, and alcohol drinking.

**Table 5:** Adjusted odds ratio (aOR) of the clustering of MetS risk components associated with beverage consumption and physical activity in adolescents, Taiwan.

Factor/Category	Male					Female				
	High BMI	High FG	High BMI or High FG	≥2 MetS-aC	MetS	High WC	Low HDL-C	High WC or Low HDL-C	≥2 MetS-aC	MetS
	aOR ^1^ (95% CI)	aOR ^1^ (95% CI)	aOR ^1^ (95% CI)	aOR ^1^ (95% CI)	aOR ^1^ (95% CI)	aOR ^1^ (95% CI)	aOR ^1^ (95% CI)	aOR ^1^ (95% CI)	aOR ^1^ (95% CI)	aOR ^1^ (95% CI)
**Type of beverage**										
**Coffee**										
≥1 vs. <1 drink/week	2.1 (1.3–3.3)	1.2 (0.8–1.9)	1.7 (1.1–2.6)	1.7 (0.9–3.0)	2.3 (0.9–6.0)	1.6 (1.0–2.6)	1.1 (0.7–1.7)	1.3 (0.8–1.9)	1.6 (0.9–2.7)	2.2 (0.7–6.8)
**Sport drink**										
1–6 vs. <1 drink/week	1.2 (0.8–1.8)	1.1 (0.7–1.6)	1.2 (0.9–1.7)	1.2 (0.7–2.0)	0.5 (0.2–1.3)	1.3 (0.7–2.3)	1.1 (0.7–2.0)	1.2 (0.7–1.9)	1.0 (0.4–2.3)	4.7 (1.3–17.0)
≥7 vs. <1 drink/week	1.9 (0.8–4.7)	3.5 (1.3–9.1)	2.8 (1.1–6.9)	2.4 (0.8–6.8)	3.4 (0.97–12.1)	0.2 (0.0–1.1)	1.2 (0.3–4.5)	0.7 (0.2–2.6)	0.3 (0.1–2.1)	1.9 (0.2–18.1)
**Soda**										
1–6 vs. <1 drink/week	0.8 (0.6–1.3)	1.4 (0.95–2.0)	1.1 (0.8–1.6)	0.8 (0.5–1.4)	0.8 (0.4–1.9)	1.1 (0.7–1.9)	1.3 (0.8–2.2)	1.3 (0.8–2.0)	1.1 (0.5–2.4)	2.5 (0.7–9.1)
≥7 vs. <1 drink/week	2.1 (0.8–5.6)	1.4 (0.5–3.7)	1.4 (0.6–3.5)	3.5 (1.2–10.0)	4.6 (1.1–19.0)	0.4 (0.1–3.0)	0.9 (0.2–3.9)	0.5 (0.1–2.1)	0.8 (0.1–6.6)	NA
**Tea**										
1–6 vs. <1 drink/week	1.0 (0.6–1.6)	1.0 (0.7–1.5)	1.1 (0.8–1.6)	1.1 (0.6–1.9)	0.6 (0.2–1.6)	1.4 (0.9–2.1)	1.1 (0.7–1.7)	1.1 (0.8–1.6)	1.8 (1.1–3.0)	3.0 (1.2–7.4)
≥7 vs. <1 drink/week	1.8 (0.98–3.2)	0.9 (0.5–1.5)	1.5 (0.9–2.5)	1.9 (0.9–3.8)	1.1 (0.4–3.2)	2.0 (1.0–3.8)	1.4 (0.7–2.6)	1.5 (0.9–2.6)	2.0 (0.8–4.8)	6.8 (1.3–36.6)
**Physical activity**										
**Sedentary behavior**										
≥60 vs. <60 min/day	1.1 (0.7–1.6)	1.0 (0.7–1.4)	1.0 (0.7–1.4)	1.1 (0.7–1.7)	1.5 (0.7–3.1)	1.2 (0.8–1.8)	0.8 (0.6–1.2)	1.0 (0.7–1.4)	1.1 (0.7–1.8)	0.3 (0.1–1.1)
**Physical exercise**										
≥30 vs. <30 min/day	1.4 (0.96–2.1)	0.9 (0.6–1.3)	1.1 (0.8–1.5)	1.4 (0.9–2.2)	1.0 (0.5–2.1)	1.4 (0.96–2.1)	1.2 (0.8–1.8)	1.4 (0.96–1.9)	1.4 (0.8–2.3)	0.8 (0.3–2.6)

**Abbreviations**: MetS, metabolic syndrome; FG, fasting glucose; BMI, body mass index; WC, waist circumference; HDL-C, High-density lipoprotein cholesterol; ≥2 MetS-aC, central adiposity + ≥1 abnormal components (aC) of MetS (MetS and its abnormal components were defined by TPA-IDF generalized criteria); NA, non-applicable due to limited sample size. ^1^ aORs were adjusted for area, age, daily energy intake, total sugar-sweetened beverage intake, puberty, physical activity, smoking, and alcohol drinking.

**Table 6:** Multivariable-adjusted odds ratio (aOR) of the clustering of MetS risk components associated with significant dietary habits in adolescents, Taiwan.

Factor/Category	Male					Female^1^		
	High BMI	High FG	High BMI or High FG	≥2 MetS-aC	MetS	High WC	≥2 MetS-aC	MetS
	aOR ^2^ (95% CI)	aOR ^2^ (95% CI)	aOR ^2^ (95% CI)	aOR ^2^ (95% CI)	aOR ^2^ (95% CI)	aOR ^2^ (95% CI)	aOR ^2^ (95% CI)	aOR ^2^ (95% CI)
**Dietary pattern**								
**Dairy products or fresh fruit ^3^**								
≥1 vs. <1 serving/week	0.6 (0.4–0.9)	0.9 (0.6–1.2)	0.7 (0.5–1.0)	0.5 (0.3–0.8)	0.7 (0.3–1.5)	0.8 (0.5–1.1)	0.5 (0.3–0.9)	0.5 (0.2–1.2)
**Type of beverage**								
**Coffee**								
≥1 vs. <1 drink/week	2.1 (1.3–3.3)	NI	1.7 (1.1–2.5)	NI	NI	1.5 (0.96–2.5)	NI	NI
**Sport drink**								
1–6 vs. <1 drink/week	NI	1.1 (0.7–1.6)	1.2 (0.8–1.7)	NI	NI	NI	NI	4.0 (1.4–12.0)
≥7 vs. <1 drink/week	NI	3.5 (1.4–9.0)	2.7 (1.1–6.6)	NI	NI	NI	NI	2.0 (0.2–22.8)
**Soda**								
1–6 vs. <1 drink/week	NI	NI	NI	0.8 (0.5–1.3)	0.8 (0.4–1.9)	NI	NI	NI
≥7 vs. <1 drink/week	NI	NI	NI	3.5 (1.2–10.0)	4.6 (1.1–18.5)	NI	NI	NI
**Tea**								
1–6 vs. <1 drink/week	NI	NI	NI	NI	NI	1.3 (0.9–2.0)	1.8 (1.1–3.0)	2.8 (1.1–7.0)
≥7 vs. <1 drink/week	NI	NI	NI	NI	NI	1.9 (0.97–3.6)	2.0 (0.9–4.8)	5.2 (1.3–20.5)

**Abbreviations**: MetS, metabolic syndrome; FG, fasting glucose; WC, waist circumference; BMI, body mass index; ≥2 MetS-aC, central adiposity + ≥1 abnormal components (aC) of MetS (MetS and its abnormal components were defined by TPA-IDF generalized criteria); NI, the variable that was not included in the multivariable-adjusted regression models. ^1^ Because no significant factors for the outcomes of ‘Low HDL-C’ and ‘High WC or Low HDL-C’ were found in Table 4 and Table 5, the related analyses were omitted here. ^2^ aORs were adjusted for area, age, daily energy intake, total sugar-sweetened beverage intake, puberty, physical activity, smoking, and alcohol drinking, as well as the covariates in the model. ^3^ The intakes of dairy products and fresh fruit were combined into a variable due to the strong correlation between the two factors.

## Supplementary Materials

The following are available online at https://www.mdpi.com/2072-6643/11/3/584/s1, Table S1: Criteria for the identification of metabolic syndrome defined by TPA, IDF, JIS-Adult, and TPA-IDF generalization.

## Abbreviation

BMI	body mass index
BP	blood pressure
DBP	diastolic blood pressure
FG	fasting glucose
FTG	fasting triglycerides
HC	hip circumference
HDL-C	high-density lipoprotein cholesterol
IDF	International Diabetes Federation
JIS-Adult	Joint Interim Statement of MetS for adults
MetS	metabolic syndrome
NAHSIT	Nutrition and Health Survey in Taiwan
NCEP-ATP III	National Cholesterol Education Program, Adult Treatment Panel III
SBP	systolic blood pressure
SSB	sugar-sweetened beverage
T2DM	type 2 diabetes mellitus
TPA	Taiwan Pediatric Association
WC	waist circumference

## References

[1] Eckel R.H., Grundy S.M., Zimmet P.Z. (2005). The metabolic syndrome. Lancet.

[2] Magnussen C.G., Koskinen J., Chen W., Thomson R., Schmidt M.D., Srinivasan S.R., Kivimaki M., Mattsson N., Kahonen M., Laitinen T. (2010). Pediatric metabolic syndrome predicts adulthood metabolic syndrome, subclinical atherosclerosis, and type 2 diabetes mellitus but is no better than body mass index alone: The bogalusa heart study and the cardiovascular risk in young finns study. Circulation.

[3] Ambrosini G.L., Oddy W.H., Huang R.C., Mori T.A., Beilin L.J., Jebb S.A. (2013). Prospective associations between sugar-sweetened beverage intakes and cardiometabolic risk factors in adolescents. Am. J. Clin. Nutr..

[4] Chan T.F., Lin W.T., Huang H.L., Lee C.Y., Wu P.W., Chiu Y.W., Huang C.C., Tsai S., Lin C.L., Lee C.H. (2014). Consumption of sugar-sweetened beverages is associated with components of the metabolic syndrome in adolescents. Nutrients.

[5] Dias Pitangueira J.C., Rodrigues Silva L., Portela de Santana M.L., Monteiro da Silva Mda C., de Farias Costa P.R., D’Almeida V., de Oliveira Assis A.M. (2014). Metabolic syndrome and associated factors in children and adolescents of a brazilian municipality. Nutr. Hosp..

[6] Johnson W.D., Kroon J.J., Greenway F.L., Bouchard C., Ryan D., Katzmarzyk P.T. (2009). Prevalence of risk factors for metabolic syndrome in adolescents: National health and nutrition examination survey (nhanes), 2001–2006. Arch. Pediatr. Adolesc. Med..

[7] Kim S., So W.Y. (2016). Prevalence of metabolic syndrome among korean adolescents according to the national cholesterol education program, adult treatment panel iii and international diabetes federation. Nutrients.

[8] Song P., Yu J., Chang X., Wang M., An L. (2017). Prevalence and correlates of metabolic syndrome in chinese children: The china health and nutrition survey. Nutrients.

[9] Gaston S.A., Tulve N.S., Ferguson T.F. (2019). Abdominal obesity, metabolic dysfunction, and metabolic syndrome in U.S. Adolescents: National health and nutrition examination survey 2011–2016. Ann. Epidemiol..

[10] Lakka T.A., Laaksonen D.E. (2007). Physical activity in prevention and treatment of the metabolic syndrome. Appl. Physiol. Nutr. Metab..

[11] Feldeisen S.E., Tucker K.L. (2007). Nutritional strategies in the prevention and treatment of metabolic syndrome. Appl. Physiol. Nutr. Metab..

[12] Albert Perez E., Mateu Olivares V., Martinez-Espinosa R.M., Molina Vila M.D., Reig Garcia-Galbis M. (2018). New insights about how to make an intervention in children and adolescents with metabolic syndrome: Diet, exercise vs. Changes in body composition. A systematic review of rct. Nutrients.

[13] Steinberger J., Daniels S.R., Eckel R.H., Hayman L., Lustig R.H., McCrindle B., Mietus-Snyder M.L., American Heart Association Atherosclerosis, Hypertension, Obesity in the Young Committee of the Council on Cardiovascular Disease in the Young, Council on Cardiovascular Nursing (2009). Progress and challenges in metabolic syndrome in children and adolescents: A scientific statement from the american heart association atherosclerosis, hypertension, and obesity in the young committee of the council on cardiovascular disease in the young; council on cardiovascular nursing; and council on nutrition, physical activity, and metabolism. Circulation.

[14] Zimmet P., Alberti K.G., Kaufman F., Tajima N., Silink M., Arslanian S., Wong G., Bennett P., Shaw J., Caprio S. (2007). The metabolic syndrome in children and adolescents—An idf consensus report. Pediatr. Diabetes.

[15] Cook S., Weitzman M., Auinger P., Nguyen M., Dietz W.H. (2003). Prevalence of a metabolic syndrome phenotype in adolescents: Findings from the third national health and nutrition examination survey, 1988–1994. Arch. Pediatr. Adolesc. Med..

[16] Lin W.T., Huang H.L., Huang M.C., Chan T.F., Ciou S.Y., Lee C.Y., Chiu Y.W., Duh T.H., Lin P.L., Wang T.N. (2013). Effects on uric acid, body mass index and blood pressure in adolescents of consuming beverages sweetened with high-fructose corn syrup. Int. J. Obes..

[17] Lee C.Y., Lin W.T., Tsai S., Hung Y.C., Wu P.W., Yang Y.C., Chan T.F., Huang H.L., Weng Y.L., Chiu Y.W. (2016). Association of parental overweight and cardiometabolic diseases and pediatric adiposity and lifestyle factors with cardiovascular risk factor clustering in adolescents. Nutrients.

[18] Lin W.T., Chan T.F., Huang H.L., Lee C.Y., Tsai S., Wu P.W., Yang Y.C., Wang T.N., Lee C.H. (2016). Fructose-rich beverage intake and central adiposity, uric acid, and pediatric insulin resistance. J. Pediatr..

[19] Kim Y., Je Y. (2018). Meat consumption and risk of metabolic syndrome: Results from the korean population and a meta-analysis of observational studies. Nutrients.

[20] Lin W.T., Lin P.C., Lee C.Y., Chen Y.L., Chan T.F., Tsai S., Huang H.L., Wu P.W., Chin Y.T., Lin H.Y. (2018). Effects of insulin resistance on the association between the circulating retinol-binding protein 4 level and clustering of pediatric cardiometabolic risk factors. Pediatr. Diabetes.

[21] Liou T.H., Huang Y.C., Chou P. (2009). Prevalence and secular trends in overweight and obese Taiwanese children and adolescents in 1991–2003. Ann. Hum. Biol..

[22] National Health Research Institutes Nutrition and Health Survey in Taiwan 2010–2011. https://www.hpa.gov.tw/Pages/List.aspx?nodeid=1773.

[23] Chen C.M., Lou M.F., Gau B.S. (2014). Prevalence of impaired fasting glucose and analysis of related factors in Taiwanese adolescents. Pediatr. Diabetes.

[24] (1993). Taiwanese Food and Nutrients Databank. https://consumer.Fda.Gov.Tw/foodanalysis/ingredients.Htm.

[25] Gau S.F., Soong W.T., Tsai W.Y., Chiu Y.N. (1997). A Chinese version of a self-administered rating scale for pubertal development. Taiwan. J. Psychiatr..

[26] The Statement of Taiwan Pediatric Association for Child and Adolescent Metabolic Syndrome Taiwan Pediatric Association, June 2016. https://www.Pediatr.Org.Tw/people/edu_info.Asp?Id=33.

[27] Alberti K.G., Eckel R.H., Grundy S.M., Zimmet P.Z., Cleeman J.I., Donato K.A., Fruchart J.C., James W.P., Loria C.M., Smith S.C. (2009). Harmonizing the metabolic syndrome: A joint interim statement of the international diabetes federation task force on epidemiology and prevention; national heart, lung, and blood institute; american heart association; world heart federation; international atherosclerosis society; and international association for the study of obesity. Circulation.

[28] Altman D.G. (1991). Practical Statistics for Medical Research.

[29] Lee C.H., Chiang S.L., Ko A.M., Hua C.H., Tsai M.H., Warnakulasuriya S., Ibrahim S.O., Sunarjo, Zain R.B., Ling T.Y. (2014). Betel-quid dependence domains and syndrome associated with betel-quid ingredients among chewers: An asian multi-country evidence. Addiction.

[30] Lee C.H., Ko A.M., Yen C.F., Chu K.S., Gao Y.J., Warnakulasuriya S., Sunarjo, Ibrahim S.O., Zain R.B., Patrick W.K. (2012). Betel-quid dependence and oral potentially malignant disorders in six asian countries. Br. J. Psychiatry.

[31] Lee C.H., Ko A.M., Yang F.M., Hung C.C., Warnakulasuriya S., Ibrahim S.O., Zain R.B., Ko Y.C. (2018). Association of dsm-5 betel-quid use disorder with oral potentially malignant disorder in 6 betel-quid endemic asian populations. JAMA Psychiatry.

[32] Morrison J.A., Friedman L.A., Wang P., Glueck C.J. (2008). Metabolic syndrome in childhood predicts adult metabolic syndrome and type 2 diabetes mellitus 25 to 30 years later. J. Pediatr..

[33] Morrison J.A., Friedman L.A., Gray-McGuire C. (2007). Metabolic syndrome in childhood predicts adult cardiovascular disease 25 years later: The princeton lipid research clinics follow-up study. Pediatrics.

[34] Magge S.N., Goodman E., Armstrong S.C., Committee On N., Section On E., Section On O. (2017). The metabolic syndrome in children and adolescents: Shifting the focus to cardiometabolic risk factor clustering. Pediatrics.

[35] Elwood P.C., Pickering J.E., Fehily A.M. (2007). Milk and dairy consumption, diabetes and the metabolic syndrome: The caerphilly prospective study. J. Epidemiol. Community Health.

[36] Fumeron F., Lamri A., Emery N., Bellili N., Jaziri R., Porchay-Balderelli I., Lantieri O., Balkau B., Marre M., Group D.S. (2011). Dairy products and the metabolic syndrome in a prospective study, desir. J. Am. Coll. Nutr..

[37] Zhang Y., Zhang D.Z. (2018). Associations of vegetable and fruit consumption with metabolic syndrome. A meta-analysis of observational studies. Public Health Nutr..

[38] Richelsen B. (2013). Sugar-sweetened beverages and cardio-metabolic disease risks. Curr. Opin. Clin. Nutr. Metab. Care.

[39] Ter Horst K.W., Serlie M.J. (2017). Fructose consumption, lipogenesis, and non-alcoholic fatty liver disease. Nutrients.

[40] Chiu S., Mulligan K., Schwarz J.M. (2018). Dietary carbohydrates and fatty liver disease: De novo lipogenesis. Curr. Opin. Clin. Nutr. Metab. Care.

[41] Low W.S., Cornfield T., Charlton C.A., Tomlinson J.W., Hodson L. (2018). Sex differences in hepatic de novo lipogenesis with acute fructose feeding. Nutrients.

[42] Rodriguez L.A., Madsen K.A., Cotterman C., Lustig R.H. (2016). Added sugar intake and metabolic syndrome in us adolescents: Cross-sectional analysis of the national health and nutrition examination survey 2005–2012. Public Health Nutr..

[43] Gui Z.H., Zhu Y.N., Cai L., Sun F.H., Ma Y.H., Jing J., Chen Y.J. (2017). Sugar-sweetened beverage consumption and risks of obesity and hypertension in Chinese children and adolescents: A national cross-sectional analysis. Nutrients.

[44] Ha K., Chung S., Lee H.S., Kim C.I., Joung H., Paik H.Y., Song Y. (2016). Association of dietary sugars and sugar-sweetened beverage intake with obesity in Korean children and adolescents. Nutrients.

[45] Department of Statistics, Taiwanese Ministry of Education. https://stats.moe.gov.tw/qframe.aspx?qno=MQA1AA2.

