# Supplementary material for: Clustering of Metabolic Risk Components and Associated Lifestyle Factors: A Nationwide Adolescent Study in Taiwan
Source: Nutrients. 2019 Mar 9;11(3):584. doi: 10.3390/nu11030584 (PMC6471895; doi:10.3390/nu11030584)
Supplement: Supplementary file 1 [file nutrients-11-00584-s001.pdf]

Supplementary table1. Criteria for the identification of metabolic syndrome defined by TPA, IDF, JIS-Adult and TPA-IDF generalization

MetS components	TPA (for 8-18 yrs) [26]	IDF (for 10-18 yrs) [14]	JIS-Adult (for Asians ≥18 yrs) [27]	TPA-IDF generalization[5] (for 12-18 yrs) [14,26]
Central obesity	BMI >95 percentile for gender-age-specific groups[1]	WC ≥90 percentile (or adult cutoff if lower)[2,3]	WC ≥90 cm in males, WC ≥80 cm in females[4]	BMI >95 percentile for gender-age-specific groups[1], or WC ≥90 percentile (or adult cutoff if lower)[2,3]
Low high-density lipoprotein cholesterol	<40 mg/dL in males, <50 mg/dL in females	<40 mg/dL (10-15 yrs); <40 mg/dL in males, <50 mg/dL in females (16-18 yr)	<40 mg/dL in males, <50 mg/dL in females	<40 mg/dL in males, <50 mg/dL in females
Increased blood pressure	SBP ≥130 / DBP ≥85 mmHg (or SBP/DBP > 95 percentile for gender-age-specific groups)	SBP ≥130 / DBP ≥85 mmHg (or antihypertensive drug treatment)	SBP ≥130 / DBP ≥85 mmHg (or antihypertensive drug treatment)	SBP ≥130 / DBP ≥85 mmHg (or SBP/DBP > 95 percentile for gender-age-specific groups, use antihypertensive drug)
Elevated triglyceride	≥150 mg/dL	≥150 mg/dL	≥150 mg/dL	≥150 mg/dL
High fasting glucose	≥100 mg/dL (or previously diagnosed type 2 diabetes)	≥100 mg/dL (or previously diagnosed type 2 diabetes)	≥100 mg/dL (or previously diagnosed type 2 diabetes)	≥100 mg/dL (or previously diagnosed type 2 diabetes)
Diagnostic criteria for MetS	**Central obesity plus 2 other risk components**	**Central obesity plus 2 other risk components**	**Any 3 or more risk components**	**Central obesity plus 2 other risk components**

Abbreviation: TPA, Taiwan Pediatric Association; IDF, International Diabetes Federation; JIS-Adult, Joint Interim Statement of MetS for adults; BMI, body mass index; WC, waist circumference; SBP, systolic blood pressure; DBP, diastolic blood pressure; HDL-C, high-density lipoprotein cholesterol.

[1]The cut-off points of central obesity for gender-age-specific adolescents are defined by TPA.

[2]There are no national data on the cutoff points of WC >90 percentile for adolescents in Taiwan. This nationwide data showed that the 90 percentiles of WC in both boys and girls were higher than the adult cutoff points, thus the adult cutoff points (WC ≥90 cm in males and ≥80 cm in females) were applied.

[3]If BMI is > 30kg/m², central obesity can be assumed, waist circumference does not need to be considered.

[4]The recommended WC thresholds of abdominal obesity for Asian adults.

[5]The is generalized to include adolescents who meet the TPA or IDF criteria.
